# The Effect of High-Density Lipoprotein on the Rheumatic Mitral Valve Calcification and Surgical Prognosis

**DOI:** 10.1016/j.jacasi.2025.07.025

**Published:** 2025-11-06

**Authors:** Peiyi Liu, Songhao Jia, Tingting Liu, Maozhou Wang, Yazhe Zhang, Jie Han, Wenjian Jiang

**Affiliations:** a Department of Cardiac Surgery, Beijing Anzhen Hospital, Capital Medical University, Beijing, China; b Beijing Lab for Cardiovascular Precision Medicine, Beijing, China; c Echocardiography Medical Center, Beijing Anzhen Hospital, Capital Medical University, China; d Laboratory for Clinical Medicine, Capital Medical University, Beijing, China

**Keywords:** high-density lipoprotein, mitral valve calcification, rheumatic heart valve disease, surgical prognosis

## Abstract

**Background:**

Rheumatic heart disease remains the most common valvular heart disease in Asia and often leads to severe calcification of the valve leaflets. However, the causes of calcification and the factors influencing the prognosis of surgical patients are not yet clearly understood.

**Objectives:**

This study aimed to explore the impact of plasma high-density lipoprotein (HDL) levels on the formation of rheumatic mitral valve calcification and the surgical prognosis.

**Methods:**

This retrospective cohort study included 847 patients who underwent mitral valve surgery for rheumatic heart disease at the Beijing Anzhen Hospital from January 2016 to December 2022. Patients were divided into a high HDL group (n = 413) and a low HDL group (n = 434) based on preoperative HDL levels.

**Results:**

Among 847 surgical patients (median follow-up 44.1 months), Kaplan-Meier analysis revealed that the high HDL group had higher mid-term survival (*P* = 0.018) and lower major adverse cardiovascular events risk (*P* = 0.005). Multivariate Cox regression confirmed HDL as independent risk factor for major adverse cardiovascular events (HR: 0.370; 95% CI: 0.220-0.620; *P* < 0.001), whereas other traditional lipid markers, including low-density lipoprotein (HR: 0.82; 95% CI: 0.54-1.23; *P* = 0.328) and triglycerides (HR: 0.65; 95% CI: 0.38-1.10; *P* = 0.107), were not statistically significant. Logistic regression indicated HDL as a protective factor against mitral valve calcification (HR: 0.01; 95% CI: 0.000-0.300; *P* = 0.009). Restricted cubic splines indicated that Agatston scores decreased with rising HDL when <1.3 mmol/L (*P* for nonlinear = 0.05; *P* for overall = 0.014).

**Conclusions:**

The findings suggest that higher preoperative HDL levels could be correlated with lighter valve calcification, enhanced mid-term survival rates, and a decreased risk of cardiovascular events.

Rheumatic heart disease is a valvular heart disease caused by rheumatic fever, typically affecting the mitral valve, leading to mitral stenosis and regurgitation.^1^, ^2^, ^3^ Surgical intervention is a crucial method for managing rheumatic mitral valve disease.^4^^,^^5^ However, the prognosis of patients undergoing surgery for rheumatic valvular disease is influenced by various factors, including the surgical approach, preoperative cardiac function, and lipid metabolism levels.^6^, ^7^, ^8^ High-density lipoprotein (HDL) plays an extremely important role in lipid metabolism, primarily functioning to transport cholesterol from peripheral tissues back to the liver, thereby helping to reduce the risk of atherosclerosis.^9^, ^10^, ^11^ An increasing number of studies have highlighted the significance of HDL in the prevention of cardiovascular diseases, particularly its protective role against atherosclerotic heart disease, which has become widely accepted.^12^ HDL is more of a biological marker that provides prognostic predictions for patients rather than a therapeutic target.

The results of previous clinical trials aimed at increasing HDL levels to reduce the risk of cardiovascular events have not been satisfactory.^13^ However, little attention has been given to the impact of HDL on the prognosis of surgical patients, particularly regarding its effects on rheumatic lesions and the prognosis of patients, with a lack of relevant research reports. Rheumatic lesions may influence the lipid metabolism of patients through mechanisms such as the release of inflammatory factors, thereby affecting patient prognosis and the severity of the lesions (eg, the degree of mitral valve fibrosis or calcification).

The aim of the current study was to evaluate the role of HDL in the prognosis and calcification formation of patients with rheumatic mitral valve disease.

## Methods

This retrospective cohort study included 886 patients who underwent mitral valve surgery for rheumatic heart disease at the Valve Surgery Center of Beijing Anzhen Hospital between January 2016 and December 2022. The study was approved by the Medical Ethics Committee of Beijing Anzhen Hospital, Capital Medical (University Ethics Approval Number KS2022064).

**Figure 1:**
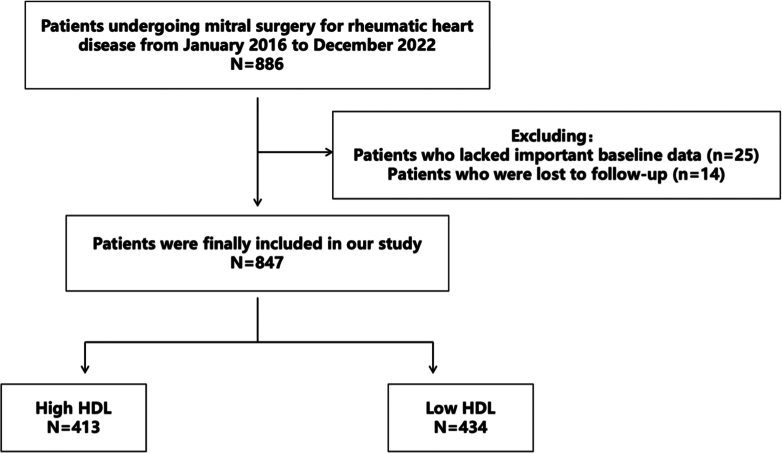
Study Design Technical flowchart of the study, which included 886 patients who underwent mitral valve surgery at the Valve Surgery Center of Beijing Anzhen Hospital from January 2016 to December 2022. A total of 25 patients with missing important baseline data and 14 lost to follow-up were excluded, resulting in 847 patients being included in the final analysis. Based on preoperative high-density lipoprotein (HDL) levels, patients were divided into the high HDL (H-HDL) group (n = 413) and the low HDL (L-HDL) group (n = 434).

Twenty-five patients were excluded who lacked important baseline data; 14 patients were also excluded who were lost to follow-up. A total of 847 patients were thus included in the study (Figure 1). All patients were diagnosed with rheumatic valvular heart disease through preoperative transthoracic echocardiography.

Patients were divided into 2 groups based on the median level of preoperative HDL: the high HDL group (H-HDL, n = 410) and the low HDL group (L-HDL, n = 434). The mid-term prognosis was compared between the 2 groups; the primary endpoint was mortality and the secondary endpoints were defined as major adverse cardiovascular events (MACE), including death, myocardial infarction, cerebral infarction, reoperation, heart failure, and recent recurrence of atrial fibrillation.

Our previous research innovatively proposed the use of coronary computed tomography (CT) imaging technology to quantify the degree of mitral valve calcification.^14^ In our study, non-contrast cardiac CT imaging was used for the quantitative assessment of mitral calcification, including both the mitral valve and sub-valvular devices, using the Agatston scoring method with semi-automatic software (VScore, Vital Images). All cardiac CT images for each patient were initially assessed by 2 trained radiology observers, with the results from the first assessment kept blinded from the second observer. The reported value was the average of the readings from both observers. In cases in which significant discrepancies were observed between the values of the first and second observers, the final value was determined through a consensus reading by the cardiologist and the radiologist.

Data were collected from the preoperative assessment and classification database for rheumatic mitral valve lesions (ChiCTR2200067151), including patient demographic characteristics, medical history, imaging, laboratory tests, and postoperative outcomes. Rheumatic heart valve disease was defined as the diagnosis of “rheumatic heart valve disease” based on preoperative transthoracic echocardiography findings. All follow-up studies were conducted through clinical or telephone follow-up contact after patient discharge, with the mid-term follow-up cutoff date set at December 12, 2022.

All surgeries were performed through a median sternotomy, with the surgical approach chosen based on the patient’s mitral valve pathology, including mitral valve repair or replacement, and concurrent tricuspid valvuloplasty, aortic valvuloplasty, aortic valve replacement, and radiofrequency ablation. All procedures were conducted with the assistance of cardiopulmonary bypass. Our research program was approved by the Ethics Committee of Beijing Anzhen Hospital (KS2022078), and the privacy and personal identifiable information of the subjects was protected.

Continuous variables with a normal distribution are presented as mean ± SD and were compared by using the Student’s *t*-test. Non-normally distributed continuous variables are expressed as median (Q1-Q3) and were compared by using the Mann-Whitney *U* test. Categorical variables are presented as n (%) and were compared by using the chi-square test or Fisher exact test, as appropriate.

Multivariable Cox regression using 3 different statistical inference models was used to investigate the association between HDL levels and mid-term mortality and the risk of cardiovascular events in patients. In Model 1, no variables were adjusted; Model 2 adjusted for age, sex, and body mass index (BMI); and Model 3 adjusted for history of coronary artery disease, history of stroke, history of infective endocarditis, mitral valve calcification score, quality of calcification, calcification volume, surgical method, preoperative creatinine levels, EuroSCORE (European System for Cardiac Operative Risk Evaluation) II, and left atrial diameter. The proportional hazards assumption was tested by using Schoenfeld residuals, and no significant violations were detected: regarding the effect of HDL on mortality, Model 1 global *P* = 0.25, Model 2 global *P* = 0.29, and Model 3 global *P* = 0.32; regarding the effect of HDL on MACE, Model 1 global *P* = 0.69, Model 2 global *P* = 0.99, and Model 3 global *P* = 0.92. This confirms that the Cox regression model was appropriate for our data. The predictive performance of each model was visualized by using time-dependent receiver-operating characteristic (ROC) curves.

In addition, restricted cubic spline (RCS) analyses were performed with 3 knots to explore the relationship between HDL levels, mitral valve calcification, and surgical outcomes. Restricted cubic splines with 4 knots were placed at clinically relevant distribution percentiles (5th, 35th, 65th, and 95th). This placement minimizes boundary bias while capturing potential nonlinearity.

For the subgroup analysis of the association between HDL and mid-term mortality as well as the risk of cardiovascular events, the data were stratified according to sex (male/female), smoking history (yes/no), alcohol consumption history (yes/no), hypertension (yes/no), coronary artery disease (yes/no), diabetes (yes/no), history of cerebral infarction (yes/no), and history of chronic obstructive pulmonary disease (yes/no), as these factors were considered potential confounders. The specific interaction between HDL and low-density lipoprotein (LDL) levels on mitral valve calcification severity was statistically nonsignificant (*P*_interaction_ = 0.42). For the remaining variables, we deemed no substantial clinical relevance for interaction analyses, which were thus not performed.

The predictive performance of each model was visualized by using time-dependent ROC curves. The prognostic accuracy assessment was fixed at the 2,000-day landmark (equivalent to 5.5 years). Bootstrap-estimated 95% CIs (500 replicates) for time-dependent ROC curves were computed by using the R survival package (version 3.5.0) with inverse probability of censoring weighting. Statistical significance was defined as a 2-sided *P* value <0.05. All analyses were conducted by using R version 4.3.2.^13^

## Results

### Perioperative Characteristics of Patients

This study included a total of 847 patients, with an average age of 58.46 ± 7.61 years. Among them, 597 were female (70.48% [597 of 847]) and 250 were male (29.52% [250 of 847]). The mean HDL level was 1.18 ± 0.29 mmol/L. In the high HDL group, there was a higher proportion of female subjects (80.63% [333 of 413] vs 60.83% [264 of 434]; *P* < 0.001), fewer patients with a history of smoking (13.32% [55 of 413] vs 23.73% [103 of 434]; *P* < 0.001), fewer patients with diabetes (7.75% [32 of 413] vs 17.05% [74 of 434]; *P* < 0.001), and fewer patients with coronary heart disease (7.02% [29 of 413] vs 11.06% [48 of 434]; *P* = 0.041). There were no significant differences between the 2 groups regarding other factors, including history of hypertension, previous cerebrovascular accident, previous heart surgery, chronic obstructive pulmonary disease, history of active endocarditis, and preoperative EuroSCORE II.

Patients in the high HDL group had lower preoperative triglyceride levels (1.18 ± 0.29 mmol/L vs 1.63 ± 0.91 mmol/L; *P* < 0.001) and higher total cholesterol levels (4.71 ± 0.96 vs 4.39 ± 0.99 mmol/L; *P* < 0.001). Preoperative transthoracic echocardiography indicated a higher left ventricular ejection fraction percentage in the high HDL group (60.69% ± 6.64% vs 59.58% ± 6.91%; *P* = 0.017). There were no significant differences between the 2 groups in other laboratory tests, including left ventricular end-diastolic diameter, end-systolic diameter, left atrial diameter, and mitral valve calcification (Agatston score). There were also no significant differences between the 2 groups in perioperative outcomes (Table 1).

**Table 1:** Baseline Characteristics According to HDL Levels

	Total (N = 847)	L-HDL (n = 434)	H-HDL (n = 413)	*P* Value
Demographic characteristics				
Age, y	58.46 ± 7.61	58.22 ± 7.63	58.70 ± 7.60	0.354
Female	597 (70.48)	264 (60.83)	333 (80.63)	**<0.001**
BMI	23.98 ± 3.19	23.90 ± 3.21	24.06 ± 3.17	0.454
History of smoking	158 (18.65)	103 (23.73)	55 (13.32)	**<0.001**
Diabetes	106 (12.51)	74 (17.05)	32 (7.75)	**<0.001**
CAD	77 (9.09)	48 (11.06)	29 (7.02)	**0.041**
Hypertension	196 (23.14)	111 (25.58)	85 (20.58)	0.085
Cerebral infarction	91 (10.74)	44 (10.14)	47 (11.38)	0.560
Previous heart surgery	48 (5.67)	19 (4.38)	29 (7.02)	0.096
COPD	74 (8.74)	45 (10.37)	29 (7.02)	0.085
Active endocarditis	2 (0.24)	2 (0.46)	0 (0.00)	0.500
EuroSCORE II	2.54 (2.05-3.34)	2.54 (2.05-3.35)	2.54 (2.09-3.41)	0.696
Laboratory test				
Creatinine, μmol/L	75.25 ± 39.55	75.65 ± 19.12	74.82 ± 53.17	0.762
Triglyceride, mmol/L	1.41 ± 0.85	1.63 ± 0.91	1.18 ± 0.71	**<0.001**
Total cholesterol, mmol/L	4.54 ± 0.98	4.39 ± 0.99	4.71 ± 0.96	**<0.001**
Imaging Characteristics				
LVEDD, mm	48.43 ± 6.12	48.39 ± 6.08	48.47 ± 6.17	0.849
LVESD, mm	32.39 ± 5.45	32.52 ± 5.27	32.27 ± 5.63	0.506
Ejection fraction, %	60.12 ± 6.80	59.58 ± 6.91	60.69 ± 6.64	**0.017**
LAD, mm	51.35 ± 9.10	51.25 ± 8.67	51.45 ± 9.54	0.749
Agatston score	14.00 (0.00- 188.00)	10.00 (0.00- 212.00)	20.00 (0.00- 177.50)	0.370
Calcium volume	18.60 (0.00- 154.15)	12.00 (0.00- 175.80)	21.35 (0.00- 148.18)	0.358
Calcium quality	3.20 (0.00- 37.35)	2.50 (0.00- 37.50)	4.75 (0.00- 36.10)	0.686

Values are mean ± SD, n (%), or median (Q1-Q3).

**Bold** values denotes statistical significance.

BMI = body mass index; CAD = coronary heart disease; COPD = chronic obstructive pulmonary disease; H-HDL = high high-density lipoprotein; HDL = high-density lipoprotein; L-HDL = low high-density lipoprotein; LAD = left atrial diameter; LVEDD = left ventricular end-diastolic diameter; LVESD = left ventricular end-systolic diameter.

### Perioperative and Mid-Term Outcomes

Table 2 presents the perioperative and mid-term outcomes for patients. In terms of perioperative outcomes, the H-HDL group had 2 patient deaths (0.49% [2 of 413]), while the L-HDL group had 7 patient deaths (1.6% [7 of 434]); there were no statistically significant difference between the 2 groups (*P* = 0.213). Regarding reoperation, 12 (2.93% [12 of 413]) patients in the H-HDL group underwent a second surgery compared with 6 (1.37% [6 of 434]) patients in the L-HDL group; again, there was no statistically significant difference between the 2 groups (1.37% [6 of 434] vs 2.93% [12 of 413]; *P* = 0.117). For other perioperative outcomes such as intensive care unit time (33.18 ± 47.65 vs 30.313 ± 37.60; *P* = 0.337), hospital stay (14.19 ± 5.13 days vs 14.30 ± 5.52 days; *P* = 0.758), ventilator time (27.89 ± 35.04 hours vs 25.24 ± 25.19 hours; *P* = 0.208), acute heart failure (2.06% [9 of 434] vs 0.73% [3 of 413]; *P* = 0.578), respiratory failure (1.83% [8 of 434] vs 0.73% [3 of 413]; *P* = 0.158), renal injury (2.06% [9 of 434] vs 0.98% [4 of 413]; *P* = 0.200), malignant arrhythmia (1.14% [5 of 434] vs 0.24% [1 of 413]; *P* = 0.250), postoperative cerebral infarction (2.97% [13 of 434] vs 3.66% [15 of 413]), severe infection (1.60% [7 of 434] vs 1.46 [6 of 413]) and secondary thoracotomy (1.83% [8 of 434] vs 2.20% [9 of 413]), there were no statistically significant differences between the 2 groups.

**Table 2:** Perioperative and Mid-Term Outcomes According to HDL Levels

	Total (N = 847)	L-HDL (n = 434)	H-HDL (n = 413)	*P* Value
Perioperative outcomes				
ICU time, h	21.00 (17.00-24.00)	21.00 (17.00-24.00)	21.00 (17.00-24.00)	0.337
Hospital stay, d	14.00 (11.00-16.00)	14.00 (11.00-16.00)	14.00 (11.00-16.00)	0.758
Ventilator time, h	19.50 (16.00-22.50)	19.50 (16.00-22.50)	19.50 (16.00-22.00)	0.208
Death	9 (1.06)	7 (1.60)	2 (0.49)	0.213
Reoperation	18 (2.13)	6 (1.37)	12 (2.93)	0.117
Acute heart failure	12 (1.42)	9 (2.06)	3 (0.73)	0.102
AKI	13 (1.53)	9 (2.06)	4 (0.98)	0.200
Respiratory failure	11 (1.30)	8 (1.83)	3 (0.73)	0.158
POCI	28 (3.31)	13 (2.97)	15 (3.66)	0.578
Arrhythmia	6 (0.71)	5 (1.14)	1 (0.24)	0.250
Severe infection	13 (1.53)	7 (1.60)	6 (1.46)	0.870
Secondary thoracotomy	17 (2.01)	8 (1.83)	9 (2.20)	0.705
Mid-term outcomes				
Death	37 (4.37)	29 (6.64)	8 (1.95)	**<0.001**
Myocardial infarction	10 (1.18)	7 (1.60)	3 (0.73)	0.393
Cerebral infarction	20 (2.36)	8 (1.83)	12 (2.93)	0.294
Reoperation	6 (0.71)	4 (0.92)	2 (0.49)	0.740
Heart failure	50 (5.90)	26 (5.95)	24 (5.85)	0.953
SMR	44 (5.19)	19 (4.35)	25 (6.10)	0.251
Atrial fibrillation	229 (27.04)	135 (30.89)	94 (22.93)	**0.009**

Values are median (Q1-Q3) or n(%).

**Bold** values denotes statistical significance.

AKI = acute kidney injury; ICU = intensive care unit; POCI = postoperative cerebral infarction; SMR = severe mitral regurgitation; other abbreviations as in Table 1.

During the follow-up period with an average duration of 44.1 months (median: 43.2 months; Q1-Q3: 20.1-63.50 months), 8 patients (1.95% [8 of 413]) in the H-HDL group died, while 29 patients (6.64% [29 of 434]) in the L-HDL group died; the mortality rate in the L-HDL group was significantly higher than that in the H-HDL group (*P* < 0.001). In addition, the incidence of postoperative atrial fibrillation in the H-HDL group was significantly lower than that in the L-HDL group (30.89% [135 of 434] vs 22.93% [94 of 413]; *P* = 0.009). For other outcome events, including postoperative myocardial infarction (1.60% [7 of 434] vs 0.74% [3 of 413]), cerebral infarction (1.83% [8 of 434] vs 2.93% [12 of 413]), heart failure (5.95% [26 of 434] vs 5.85% [24 of 413]), reoperation (0.92% [4 of 434] vs 0.49% [2 of 413]), and severe mitral regurgitation (4.35% [19 of 434] vs 6.10% [25 of 413]), there were no significant differences between the 2 groups.

### Correlation Between HDL and the Risks of Mortality

The Cox proportional hazards model was used to conduct a preliminary exploration of factors affecting postoperative mortality in patients. Univariate and multivariate Cox regression analyses were conducted by using HDL as a continuous variable; we found that age (HR: 1.11; 95% CI: 1.060-1.710; *P* < 0.001) and preoperative creatinine levels (HR: 1.01 mg/ml; 95% CI: 1.010-1.010; *P* = 0.033) were independent risk factors for increased postoperative mortality, whereas HDL demonstrated a protective effect (HR: 0.32; 95% CI: 0.09-1.10; *P* = 0.071) (Supplemental Table 1). In univariate Cox regression analyses, traditional lipid markers, including LDL (HR: 0.82; 95% CI: 0.54-1.23; *P* = 0.328) and triglycerides (HR: 0.65; 95% CI: 0.38-1.10; *P* = 0.107), showed no statistically significant associations with postoperative mortality risk.

Based on a theory-driven Cox regression approach, variables were incorporated that were hypothesized to potentially influence outcome events (eg, age, history of cardiac disease, EuroSCORE II, creatinine, triglyceride levels, HDL, LDL, total cholesterol) into the multivariate model. The analysis revealed that HDL (HR: 0.57; 95% CI: 0.15-2.10; *P* = 0.395), LDL (HR: 2.11; 95% CI: 0.74-6.60; *P* = 0.155), and triglyceride (HR: 0.44; 95% CI: 0.17-1.12; *P* = 0.086) levels were not predictive factors for mortality (Supplemental Table 1).

Because HDL is typically not targeted for drug treatment in clinical practice but is more commonly used as a biological marker for indicating patient prognosis, we aimed to better illustrate its role in identifying at-risk populations. To achieve this, patients were divided into H-HDL and L-HDL groups based on the median HDL levels of the enrolled population, and a Cox regression analysis was conducted accordingly. In the unadjusted model (HR: 0.436; 95% CI: 0.216-0.883; *P* = 0.021) and the minimally adjusted model (HR: 0.421; 95% CI: 0.205-0.865; *P* = 0.019), HDL had a significant protective effect. After full adjustment, the positive correlation between HDL levels and mid-term survival rates remained consistent (HR: 0.340; 95% CI: 0.159-0.729; *P* = 0.006), indicating that the risk of death for patients in the H-HDL group is 34% lower than that of the L-HDL group (Table 3).

**Table 3:** Multivariable Analysis to Assess the Independent Impact of HDL on Death and MACE

	Model 1		Model 2		Model 3	
	HR (95% CI)	*P* Value	HR (95% CI)	*P* Value	HR (95% CI)	*P* Value
All-cause death						
HDL (continuous)	0.324 (0.095-1.102)	0.071	0.352 (0.109-1.133)	0.08	0.269 (0.080-0.904)	0.034
L-HDL	Reference		Reference		Reference	
H-HDL	0.436 (0.216-0.883)	0.021	0.421 (0.205-0.865)	0.019	0.340 (0.159-0.729)	0.006
MACE						
HDL (continuous)	0.385 (0.244-0.607)	<0.001	0.388 (0.246-0.613)	<0.001	0.406 (0.254-0.650)	<0.001
L-HDL	Reference		Reference		Reference	
H-HDL	0.706 (0.552-0.904)	0.006	0.708 (0.551-0.911)	0.007	0.743 (0.575-0.960)	0.023

Model 1: unadjusted. Model 2: adjusted for age, gender, and BMI. Model 3: adjusted for Model 2 plus history of coronary atherosclerotic heart disease, cerebral infarction, active endocarditis, Agatston score, surgical technique, creatinine, EuroSCORE II, and LAD.

MACE = major adverse cardiovascular events; other abbreviations as in Table 1.

**Figure 2:**
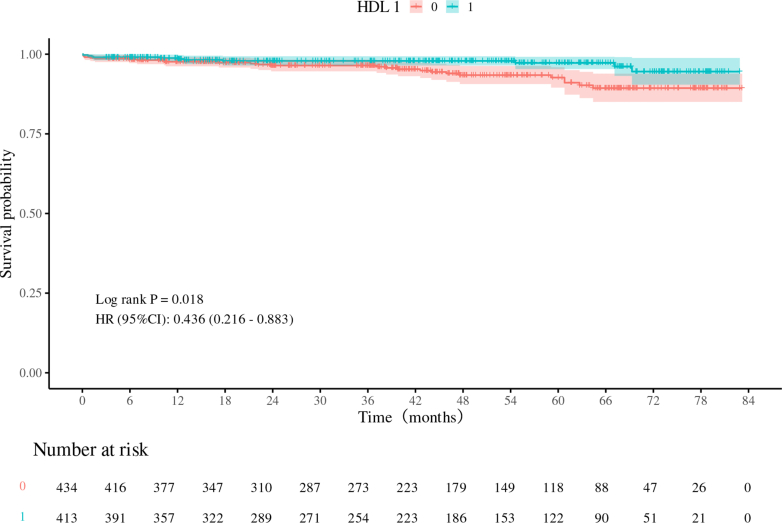
Kaplan-Meier Curve in the H-HDL Group and L-HDL Group Comparison of survival rates of patients in the H-HDL group (blue line) and the L-HDL group (red line). Survival data were obtained from 847 patients who underwent mitral valve surgery at Beijing Anzhen Hospital. Kaplan-Meier analysis shows that the median survival time in the H-HDL group was significantly longer than that in the L-HDL group (*P* = 0.018). All data were compared by using the log-rank test. Abbreviations as in Figure 1.

Figure 2 shows the differences in mid-term survival rates between patients in the H-HDL and L-HDL groups. The results indicate that the mid-term survival rate of patients in the H-HDL group was significantly better than that of the L-HDL group (HR: 0.436; 95% CI: 0.216-0.883; *P* = 0.018).

### Correlation Between HDL and the Risks of MACE

After adjusting for confounding factors such as sex, age, BMI, and past medical history, HDL had a significant protective effect on the risk of MACE (HR: 0.406; 95% CI: 0.254-0.650; *P* < 0.001) (Table 3), with each unit increase in HDL associated with a 40.6% reduction in MACE risk. Univariate and multivariate Cox regression analyses found that HDL is a protective factor against MACE occurring postoperatively (HR: 0.37; 95% CI: 0.220-0.620; *P* < 0.001) (Supplemental Table 2). Other traditional lipid markers, including LDL (HR: 0.98; 95% CI: 0.65-1.48; *P* = 0.920) and triglycerides (HR: 0.65; 95% CI: 0.38-1.10; *P* = 0.107), exhibited no statistically significant associations with postoperative MACE risk.

Patients were divided into H-HDL and L-HDL groups according to the median HDL levels (as discussed earlier), and a Cox regression analysis was conducted accordingly. In the unadjusted model (HR: 0.706; 95% CI: 0.552-0.904; *P* = 0.006) and the minimally adjusted model (HR: 0.708; 95% CI: 0.551-0.911; *P* = 0.007), HDL was a protective factor for MACE events. After full adjustment, the negative correlation between HDL levels and MACE risk remained consistent (HR: 0.743; 95% CI: 0.575-0.960; *P* = 0.023) (Table 3).

**Figure 3:**
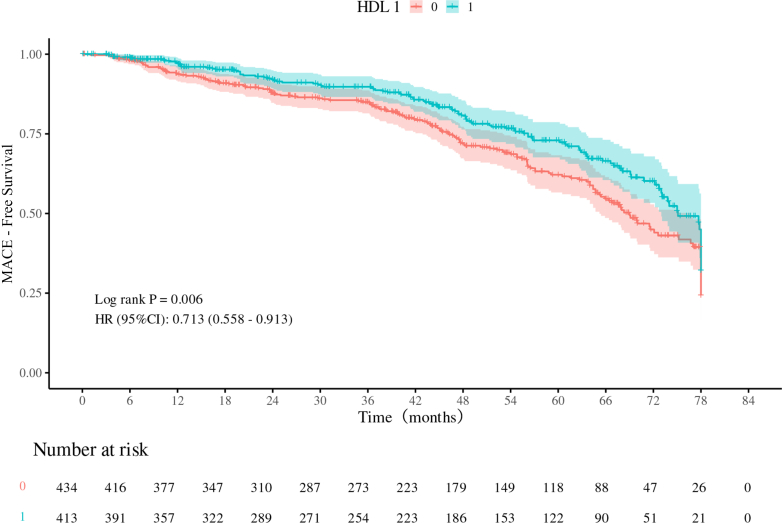
Kaplan-Meier Curve in MACE Between H-HDL Group and L-HDL Group Graphic illustrates the differences in major adverse cardiovascular events (MACE) risk between the H-HDL (blue line) and L-HDL (red line) groups. Kaplan-Meier analysis shows that the mid-term risk of MACE events is significantly lower in the H-HDL group compared with the L-HDL group (HR: 0.713; log rank test, *P* = 0.006). All data were compared by using the log-rank test. Abbreviations as in Figure 1.

**Figure 4:**
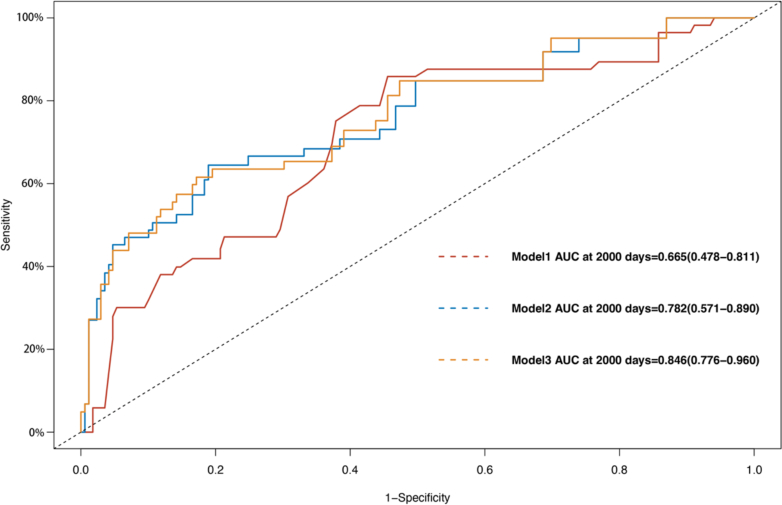
ROC Curves of Death for Three Different Models Graphic shows the predictive performance of different models for the mortality risk in patients after rheumatic surgery. Model 1: unadjusted for covariates; Model 2: adjusted for age, sex, and body mass index; and Model 3: adjusted for age, sex, body mass index, history of coronary atherosclerotic heart disease, cerebral infarction, active endocarditis, Agatston score, surgical technique, creatinine, EuroSCORE II, and left atrial diameter. AUC = area under the curve; ROC = receiver-operating characteristic.

**Figure 5:**
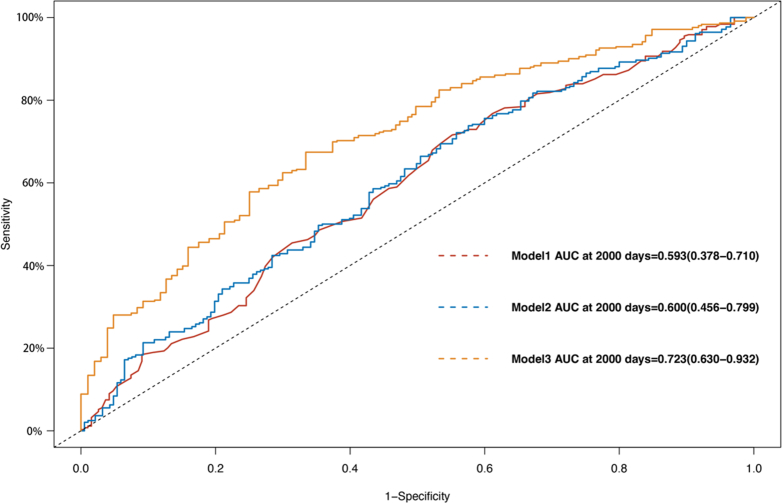
ROC Curves of MACE for Three Different Models Graphic shows the predictive performance of different models for the MACE events in patients after rheumatic surgery. Model 1: unadjusted for covariates; Model 2: adjusted for age, sex, and BMI; and Model 3: adjusted for age, sex, BMI, history of coronary atherosclerotic heart disease, cerebral infarction, active endocarditis, Agatston score, surgical technique, creatinine, EuroSCORE II, and left atrial diameter. Abbreviations as in Figures 3 and 5.

Figure 3 illustrates the differences in MACE risk between the H-HDL and L-HDL groups, with results showing that the mid-term risk of MACE events was significantly lower in the H-HDL group compared with the L-HDL group (HR: 0.713; 95% CI: 0.558-0.913; *P* = 0.006). Subsequently, ROC curves were plotted for model 1, model 2, and model 3 to illustrate the predictive performance of different models for the outcome events (Figures 4 and 5). In these 2 figures, as the number of adjusted slope variables increases, the area under the curve (AUC) gradually increases (Figure 4) (Model 1 AUC: 0.665 [95% CI: 0.478-0.811]; Model 2 AUC: 0.782 [95% CI: 0.571-0.890]; Model 3 AUC: 0.846 [95% CI: 0.776-0.960]) (Figure 5) (Model 1 AUC: 0.593 [95% CI: 0.378-0.710]; Model 2 AUC: 0.600 [95% CI: 0.456-0.799]; Model 3 AUC: 0.723 [95% CI: 0.630-0.932]). These findings indicate that HDL serves as a predictive variable with good predictive performance for the prognosis of patients after rheumatic surgery.

**Figure 6:**
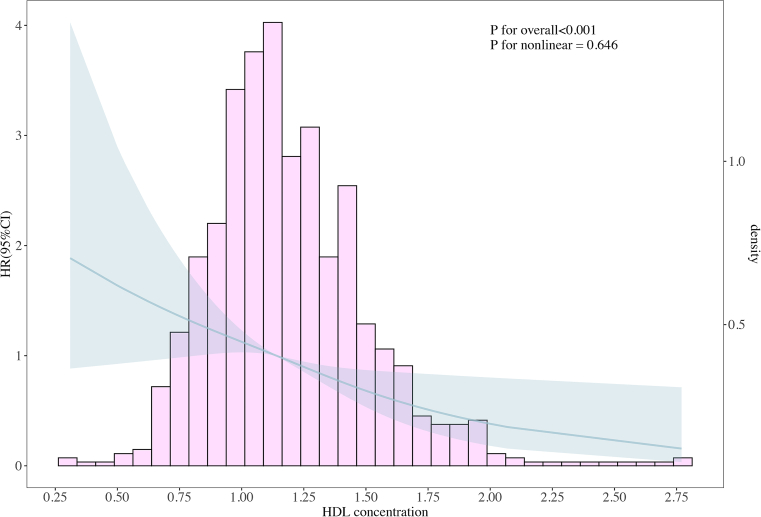
RCS Curve of HDL Levels and the Risk of MACE Graphic illustrates the nonlinear relationship between HDL levels and the risk of MACE. The data were obtained from 847 patients who underwent mitral valve surgery at Beijing Anzhen Hospital. Restricted cubic spline (RCS) analysis shows an approximately linear relationship between HDL levels and the risk of MACE (*P* for overall < 0.001; *P* for nonlinear = 0.646). The solid line represents the RCS curve, and the shaded area indicates the 95% CI. Abbreviations as in Figures 1 and 3.

RCS analyses were used to assess the potential nonlinear relationship between HDL and the risks of mortality and MACE events. The results indicate an approximately linear relationship between HDL levels and the risk of MACE (*P* for overall < 0.001; *P* for nonlinear = 0.646) (Figure 6).

### HDL and the Degree of Mitral Valve Calcification

Among the 847 patients with rheumatic valvular disease enrolled in the study, 469 patients (55.4% [469 of 847]) exhibited significant calcification of the mitral valve. This study collected coronary CT angiography images from this subset of patients and used the Agatston score, previously used to evaluate coronary calcification, to quantify the degree of mitral valve calcification. By comparing the Agatston scores, calcification volumes, and calcification qualities between the H-HDL and L-HDL groups, it was found that patients in the H-HDL group had lighter calcification compared with those in the L-HDL group, as evidenced by lower Agatston scores (163.00 vs 134.50; *P* = 0.01), calcification volumes (116.00 vs 110.45; *P* = 0.01), and calcification qualities (32.70 vs 27.70; *P* = 0.025) (Table 4).

**Table 4:** Degree of Mitral Valve Calcification Between L-HDL Group and H-HDL Group

	Total (N = 469)	L-HDL (n = 237)	H-HDL (n = 232)	*P* Value
Agatston score	142.00 (44.00-526.00)	163.00 (48.00-628.00)	134.50 (36.75-402.50)	**0.010**
Calcium volume	122.60 (39.50-424.50)	116.00 (38.15-330.02)	110.45 (31.98-322.73)	**0.010**
Calcium quality	29.50 (9.90-114.40)	32.70 (10.60-147.80)	27.70 (8.45-97.72)	**0.025**

Values are median (Q1-Q3).

**Bold** values column denotes statistical significance.

Abbreviations as in Table 1.

Table 5 presents the results of univariate and multivariate logistic regression analyses regarding the relationship between HDL levels and the degree of mitral valve calcification. The degree of mitral valve calcification was categorized into mild and severe based on a mitral Agatston score >2,000. In the univariate analysis, gender (HR: 6.07; 95% CI: 2.15-17.19; *P* < 0.001) and history of coronary artery disease (HR: 3.87; 95% CI: 1.32-11.33; *P* = 0.013) exhibited statistically significant associations. HDL levels were significantly negatively correlated with mitral valve calcification (HR: 0.09; 95% CI: 0.01-0.58; *P* = 0.012), while other variables (eg, age, BMI, diabetes) did not reach statistical significance (*P* > 0.05).

**Table 5:** HDL and Mitral Valve Calcification: Univariate/Multivariate Logistic Regression

	Univariate Analysis		Multivariate Analysis	
	HR (95% CI)	*P* Value	HR (95% CI)	*P* Value
Age	1.02 (0.96-1.08)	0.513	1.03 (0.97-1.09)	0.410
Sex	6.07 (2.15-17.19)	0.001	0.20 (0.07-0.58)	0.003
BMI	1.00 (0.87-1.16)	0.962		
CAD	3.87 (1.32-11.33)	0.013		
Diabetes	1.22 (0.34-4.31)	0.759	0.49 (0.06-3.84)	0.497
Hypertension	0.38 (0.09-1.68)	0.997		
COPD	1.92 (0.54-6.88)	0.315		
History of alcohol consumption	1.91 (0.67-5.46)	0.229		
HDL	0.09 (0.01-0.58)	0.012	0.14 (0.02-1.10)	0.042
LDL	1.19 (0.71-1.97)	0.514	0.92 (0.52-1.62)	0.768
Total cholesterol	1.01 (0.64-1.58)	0.947		
Blood glucose	1.00 (0.71-1.42)	0.997		
Triglyceride	1.21 (0.69-2.12)	0.509		

LDL = low-density lipoprotein; other abbreviations as in Table 1.

After adjusting for covariates in the multivariate analysis model, HDL and gender maintained a strong negative association (HR: 0.14 [95% CI: 0.02-1.10; *P* = 0.042]; HR: 0.20 [95% CI: 0.07-0.58; *P* = 0.003]). Other covariates, such as age (HR: 1.03; 95% CI: 0.97-1.09; *P* = 0.410), diabetes (HR: 0.49; 95% CI: 0.06-3.84; *P* = 0.497), and LDL (HR: 0.92; 95% CI: 0.52-1.62; *P* = 0.768) did not show significant associations, and variables such as gender and BMI were not included in the final multivariate model. The results suggest that HDL may be an independent protective factor for the degree of mitral valve calcification, remaining highly significant even after adjustment for potential confounding factors such as age and diabetes.

**Figure 7:**
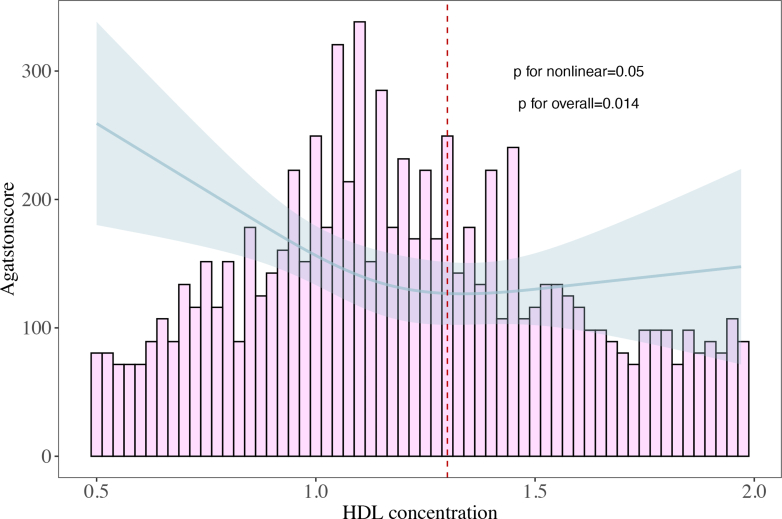
RCS Curve of HDL and Degree of Mitral Valve Calcification Graphic illustrates the nonlinear relationship between HDL levels and Agatston score of mitral valve calcification. The data were obtained from 469 patients who underwent mitral valve surgery at Beijing Anzhen Hospital with significant mitral valve calcification. RCS analysis showed that when HDL levels were <1.3 mmol/L, the degree of mitral valve calcification decreased as plasma HDL levels increased. The solid line represents the RCS curve, and the shaded area indicates the 95% CI. Abbreviations as in Figures 1, 3, and 6.

Restrictive cubic spline analysis indicated a significant nonlinear relationship between HDL levels and the degree of mitral valve calcification (Agatston score) (*P* for overall = 0.014; *P* for nonlinear = 0.05). Specifically, when HDL levels were <1.3 mmol/L, the degree of mitral valve calcification decreased as plasma HDL levels increased (Figure 7).

### Subgroup Analysis

In the subgroup analysis and interaction tests, we examined the relationship between HDL levels and the risks of mortality and MACE across different population subgroups. Among female patients (HR: 0.29; 95% CI: 0.11-0.73; *P* = 0.009) and patients with no history of alcohol consumption (HR: 0.36; 95% CI: 0.17-0.79; *P* = 0.01), diabetes (HR: 0.42; 95% CI: 0.20-0.88; *P* = 0.022), stroke (HR: 0.42; 95% CI: 0.19-0.92; *P* = 0.03), or chronic obstructive pulmonary disease (HR: 0.43; 95% CI: 0.21-0.91), the risk of mid-term mortality was lower (Supplemental Figure 1).

For mid-term MACE risk, female patients (HR: 0.70; 95% CI: 0.52-0.94; *P* = 0.017) and patients with no history of alcohol consumption (HR: 0.73; 95% CI: 0.56-0.95; *P* = 0.02), diabetes (HR: 0.72; 95% CI: 0.56-0.94; *P* = 0.014), coronary heart disease (HR: 0.70; 95% CI: 0.54-0.91; *P* = 0.007), hypertension (HR: 0.59; 95% CI: 0.44-0.79; *P* < 0.001), cerebral infarction (HR: 0.73; 95% CI: 0.56-0.94; *P* = 0.017), or chronic obstructive pulmonary disease (HR: 0.70; 95% CI: 0.54-0.91; *P* = 0.008) also had a lower risk of mid-term MACE events (Supplemental Figure 2).

## Discussion

The main findings of this study are presented in the Central Illustration. We discovered that in rheumatic mitral valve patients undergoing cardiac surgery, higher levels of HDL serve as a protective factor predicting favorable mid-to-long-term prognosis. In addition, we observed a complex nonlinear relationship between HDL levels and the degree of calcification in rheumatic mitral valves. However, the precise underlying mechanisms remain unclear. We speculate this process may be similar to how HDL mitigates the severity of coronary atherosclerosis. We found that HDL serves as a protective factor for mid-term survival and the prevention of MACE in these patients. Kaplan-Meier survival curve analysis revealed that the mid-term survival rate of the H-HDL group was significantly higher than that of the L-HDL group (HR: 0.436; *P* = 0.018). Furthermore, after adjusting for age, sex, BMI, and medical history, the Cox proportional hazards model indicated that HDL is an independent risk factor for surgical prognosis. Contrary to their established role in atherosclerotic cardiovascular disease, LDL and triglycerides did not predict mortality or MACE outcomes in this cohort. This divergence may reflect distinct pathophysiological mechanisms in rheumatic mitral valve disease. RCS analysis also showed a linear relationship between HDL levels and the risk of postoperative MACE events.

**Central Illustration:**
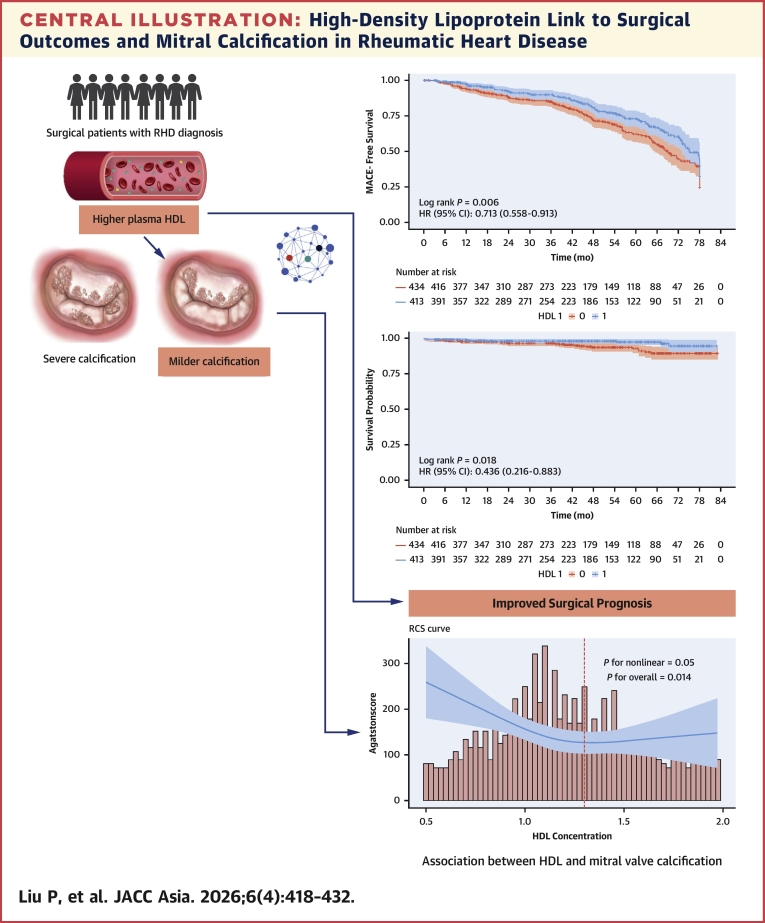
High-Density Lipoprotein Link to Surgical Outcomes and Mitral Calcification in Rheumatic Heart Disease Graphic summarizes the core findings of this study: the correlation between high-density lipoprotein (HDL) levels and both surgical outcomes and the degree of mitral valve calcification in patients with rheumatic heart disease (RHD). The two Kaplan-Meier curves in the upper right quadrant show superior outcomes regarding mortality and major adverse cardiovascular events (MACE) in patients with higher HDL levels compared with those with lower levels. The lower panel displays pathologic specimens representing varying degrees of mitral valve calcification, alongside a graphical plot illustrating the nonlinear correlation between HDL concentrations and calcification severity.

Upon further investigation, we found that among patients with valve calcification, those in the H-HDL group had milder calcification compared with the L-HDL group. In addition, there was a significant nonlinear relationship between HDL levels and the degree of mitral valve calcification. The degree of calcification typically suggests a longer medical history and poorer cardiac function in patients, which may be one of the reasons for the observed differences in survival rates and risks of MACE events between the 2 groups.

HDL is widely recognized as a “good lipoprotein.” The Framingham study conducted in the 1970s found that HDL may play a crucial role in the prevention and treatment of coronary artery disease, with levels inversely related to atherosclerosis.^13^, ^14^, ^15^, ^16^ Previous studies on lipoproteins and their impact on heart valve diseases have primarily focused on their relationship with aortic valve calcification.^17^ Research by Li et al^18^ discussed how elevated levels of remnant cholesterol may promote calcification of the aortic valve, thereby increasing the risk of aortic valve stenosis. Studies by Juris Hofmanis et al^19^ indicated that HDL-C levels are associated with the progression of aortic stenosis, particularly noting that HDL-C levels correlate with disease severity in female patients with aortic stenosis. However, there has been little research focusing specifically on the effects of HDL on the degree of rheumatic mitral valve disease and surgical prognosis.

Research has shown that the primary pathologic process of rheumatic injury is the chronic inflammatory damage to vascular endothelial cells beneath the connective tissue matrix.^20^ It is well known that during the process of atherosclerosis, the activation of inflammatory cells leads to the destruction of endothelial cells, resulting in the accumulation of intracellular lipid components within the vessels, which contributes to atherosclerosis. Based on this, we theorized that damage to valvular endothelial cells may also lead to lipid accumulation, resulting in rheumatic changes in the valvular tissue. Xie et al^21^ were the first to discover adipose tissue in rheumatic valve tissue and suggested that there may be a conversion of valvular fibroblasts and vascular matrix cells into adipocytes during the inflammatory regulation process. This study also highlighted the important role of macrophages in the occurrence and progression of rheumatic diseases. A retrospective cohort study by Antonini-Canterin et al^22^ found that the use of statins can slow the progression of rheumatic aortic valve sclerosis and mild aortic stenosis, which is somewhat consistent with the current study results. However, HDL is not a treatment target, and statins are primarily used to lower LDL levels.

The current study found that LDL is not a predictor of prognosis in patients undergoing rheumatic surgery. However, although HDL is not a therapeutic target for lipid-lowering drugs, it can indicate the degree of mitral valve calcification and the surgical outcomes. We therefore believe that the clinical significance of HDL in rheumatic mitral valve disease is not as a target for drug therapy but rather more as a biomarker that indicates the extent of calcification and prognosis risk in patients. Further research is thus needed to explore the relationship between specific lipid components and the occurrence, progression, and exacerbation of rheumatic valvular disease.

Patients with rheumatic mitral valve disease typically have a long disease course, as rheumatic fever chronically affects the mitral valve, resulting in significant valvular pathology, including leaflet thickening, retraction, and calcification,^23^ often accompanied by involvement of the sub-valvular structures. Current guidelines on the surgical treatment of rheumatic mitral valve disease remain controversial.^7^^,^^24^, ^25^, ^26^ Previous studies from our center have shown that patients undergoing mitral valve repair for rheumatic disease have significantly better mid-term survival rates and lower rates of major cardiovascular adverse events compared with those undergoing mitral valve replacement.^8^ We also found that the degree of mitral valve calcification is a major factor influencing the success rate of repair.^14^ The greater the degree of mitral valve calcification, the higher the rate of repair failure, leading to poorer outcomes for patients. Therefore, it is particularly important to comprehensively assess the degree of mitral valve calcification using preoperative ultrasound, CT imaging, and laboratory tests.

Mitral valves affected by rheumatic disease tend to develop calcification as the disease progresses, which is often associated with poor hemodynamic conditions and the long-term impact of rheumatic fever on the valve. However, no studies have yet confirmed whether abnormalities in lipid metabolism exacerbate the degree of mitral valve calcification. In the current study, approximately 55.4% of patients with rheumatic valvular disease exhibited varying degrees of mitral valve calcification. Patients in the H-HDL group had significantly lower degrees of mitral valve calcification compared with those in the L-HDL group. In addition, analysis revealed a significant nonlinear relationship between HDL and mitral valve calcification scores. Considering the pathologic features of rheumatic mitral valve disease, we hypothesize that higher levels of HDL typically indicate a mild degree of mitral valve calcification. The specific pathophysiological mechanisms remain unclear, but we believe this process may be similar to its role in reducing atherosclerosis, as the end result of atherosclerosis is often calcification of the vessel wall and plaques.

At the same time, the current study found that higher levels of HDL are a protective factor for patient prognosis, which is consistent with previous research on coronary artery disease. We hypothesize that HDL may influence mid-term outcomes by alleviating the degree of coronary atherosclerosis in patients. To address the aforementioned issues, we included patients’ medical history (with a focus on the history of coronary heart disease) as an important covariate in the Cox proportional hazards model. The results indicated that, after adjusting for past medical history in the multivariate Cox regression model, HDL levels remained an independent risk factor. Therefore, we believe that HDL is an independent risk factor affecting patients’ mid-term survival rates and the risk of MACE.

### Study Limitations

The function of HDL is not solely dependent on its concentration but is also closely related to its composition and functional characteristics. For example, the size, shape, and types of proteins and lipids carried by HDL particles may all influence their role in cardiovascular protection. However, this study was a retrospective case-control study, and due to the limitations in collecting clinical data, we were unable to explore in detail the impact of different HDL molecules on prognosis. Future research should focus more on the functional state of HDL rather than just its concentration to more comprehensively assess its impact on the surgical prognosis of patients with rheumatic mitral valve disease.

In addition, this study was a single-center investigation, which may affect the generalizability of the results. However, as the largest cardiac center in the country, this institution can somewhat mitigate the impact of regional differences. Nevertheless, future larger scale, multicenter prospective studies are needed to further validate the role of HDL in the surgical prognosis of patients with rheumatic mitral valve disease.

## Conclusions

HDL plays an important role in reducing the formation of rheumatic mitral valve calcification and improving patient outcomes. By gaining a deeper understanding of the mechanisms and functions of HDL, we may be able to provide better strategies for the management and treatment of these patients, ultimately improving their clinical outcomes.

## Funding Support and Author Disclosures

This study was supported by Noncommunicable Chronic Diseases-National Science and Technology Major Project (2023ZD0514000), the National Key R&D Program of China (2021YFC2501104, 2022YFE0209800), National Science Foundation of China (82422007, 82241205, 82170487), Beijing Natural Science Foundation (JQ24039, 7244326), Beijing Anzhen Hospital Major Science and Technology Innovation Fund (KCZD202203, KCQY202201) and Talent development plan for the future in Medical-Engineering Integration by BRA-CDCHE and ZTA. The authors have reported that they have no relationships relevant to the contents of this paper to disclose.
