# Supplementary material for: The Effect of High-Density Lipoprotein on the Rheumatic Mitral Valve Calcification and Surgical Prognosis
Source: JACC Asia. 2025 Nov 6;6(4):418–32. doi: 10.1016/j.jacasi.2025.07.025 (PMC13080751; doi:10.1016/j.jacasi.2025.07.025)
Supplement: Supplementary Tables 1 and 2 and Supplementary Figures 1 and 2 [file mmc1.docx]

Supplemental Table 1. Univariate and multivariate Cox proportional hazards analysis of independent risk factors for death.

| Variables | Univariate Analysis | | Multivariate Analysis | |
| --- | --- | --- | --- | --- |
|  | HR（95% CI） | P | HR（95% CI） | P |
| Age | 1.13 (1.07 ~ 1.18) | <0.001 | 1.11 (1.06 ~ 1.17) | **<0.001** |
| Gender | 0.75 (0.38 ~ 1.48) | 0.414 |  |  |
| BMI | 1.07 (0.97 ~ 1.18) | 0.168 |  |  |
| Smoke | 1.33 (0.63 ~ 2.84) | 0.454 |  |  |
| Diabetes | 0.67 (0.24 ~ 1.91) | 0.456 |  |  |
| CAD | 1.50 (0.53 ~ 4.25) | 0.447 |  |  |
| Hypertension | 1.18 (0.57 ~ 2.44) | 0.658 |  |  |
| Cerebral infraction | 1.52 (0.62 ~ 3.70) | 0.360 |  |  |
| PHS | 1.07 (0.26 ~ 4.46) | 0.925 | 0.65(0.13 ~ 3.25) | 0.598 |
| COPD | 1.48 (0.52 ~ 4.20) | 0.460 |  |  |
| AE | 0.00 (0.00 ~ Inf) | 0.997 |  |  |
| Agatston score | 1.01 (1.01 ~ 1.01) | 0.016 | 1.00 (1.00 ~ 1.01) | 0.136 |
| EuroSCOREII | 1.19 (1.06 ~ 1.33) | 0.004 | 1.09 (0.94 ~ 1.26) | 0.255 |
| Creatinine | 1.01 (1.01 ~ 1.01) | 0.025 | 1.00 (1.00 ~ 1.01) | **0.025** |
| Triglyceride | 0.65 (0.38 ~ 1.10) | 0.107 | 0.43 (0.17 ~ 1.12) | 0.437 |
| HDL | 0.32 (0.09 ~ 1.10) | 0.071 | 0.57 (0.15 ~ 2.10) | 0.395 |
| LDL | 0.82 (0.54 ~ 1.23) | 0.328 | 2.11 (0.74 ~ 6.60) | 0.155 |
| Total Cholesterol | 0.72 (0.51 ~ 1.01) | 0.060 | 0.44 (0.17 ~ 1.12) | 0.086 |
| LVEDD | 1.04 (0.99 ~ 1.09) | 0.163 |  |  |
| LVESD | 1.03 (0.98 ~ 1.09) | 0.225 |  |  |
| EF | 1.00 (0.95 ~ 1.05) | 0.992 |  |  |
| LAD | 1.04 (1.01 ~ 1.07) | 0.014 | 1.03（1.00～1.07） | 0.09 |

BMI, Body Mass Index; CAD, Coronary Heart Disease; COPD, Chronic Obstructive Pulmonary Disease; LVEDD, Left Ventricular End-Diastolic Diameter; LVESD, Left Ventricular End Systolic Diameter; EF, Ejection Fraction; LAD, Left Atrial Diameter; CV, Calcium volume; CQ, Calcium quality; PHS, Previous heart surgery; CI, Cerebral infarction; AE, Active endocarditis.

Supplemental Table 2. Univariate and multivariate Cox proportional hazards analysis of independent risk factors for MACE.

| Variables | Univariate Analysis | | Multivariate Analysis | |
| --- | --- | --- | --- | --- |
|  | HR（95% CI） | P | HR（95% CI） | P |
| Age | 1.00 (0.99 ~ 1.02) | 0.627 | 1.00 (0.99 - 1.02) | 0.526 |
| Gender | 0.86 (0.66 ~ 1.12) | 0.276 |  |  |
| BMI | 1.01 (0.98 ~ 1.05) | 0.455 |  |  |
| Smoke | 0.87 (0.63 ~ 1.19) | 0.329 |  |  |
| Diabetes | 0.72 (0.50 ~ 1.04) | 0.080 |  |  |
| CAD | 1.56 (1.05 ~ 2.33) | 0.028 | 1.45 (0.96 - 2.18) | 0.079 |
| Hypertension | 1.08 (0.82 ~ 1.42) | 0.600 |  |  |
| Cerebral infarction | 1.01 (0.69 ~ 1.49) | 0.944 |  |  |
| Previous heart surgery | 1.00 (0.68 ~ 1.47) | 0.981 | 1.34 (0.76 - 2.33) | 0.293 |
| COPD | 1.08 (0.70 ~ 1.66) | 0.735 |  |  |
| Active endocarditis | 0.00 (0.00 ~ Inf) | 0.933 |  |  |
| Agatston score | 1.01 (1.01 ~ 1.01) | 0.055 | 1.00 (1.00 - 1.00) | 0.088 |
| EuroSCORE II | 0.95 (0.87 ~ 1.02) | 0.158 | 0.92 (0.84 - 1.00) | 0.052 |
| Creatinine | 1.00 (1.00 ~ 1.00) | 0.175 | 1.00 (0.99 - 1.00) | 0.430 |
| Triglyceride | 1.02 (0.88 ~ 1.17) | 0.819 | 1.05 (0.73 - 1.50) | 0.794 |
| HDL | 0.39 (0.25 ~ 0.62) | <0.001 | 0.37(0.22~0.62) | <0.001 |
| LDL | 1.00 (0.86 ~ 1.15) | 0.948 | 0.98 (0.65 - 1.48) | 0.920 |
| Total Cholesterol | 0.95 (0.84 ~ 1.07) | 0.401 |  |  |
| LVEDD | 1.00 (0.98 ~ 1.02) | 0.820 |  |  |
| LVESD | 1.00 (0.98 ~ 1.03) | 0.746 |  |  |
| EF | 0.99 (0.97 ~ 1.00) | 0162 |  |  |
| LAD | 1.00 (0.98 ~ 1.01) | 0.634 |  |  |

BMI, Body Mass Index; CAD, Coronary Heart Disease; COPD, Chronic Obstructive Pulmonary Disease; LVEDD, Left Ventricular End-Diastolic Diameter; LVESD, Left Ventricular End Systolic Diameter; EF, Ejection Fraction; LAD, Left Atrial Diameter; CV, Calcium volume; CQ, Calcium quality.

Supplemental Figure 1. The impact of high-density lipoprotein on mortality risk in different subgroups of the population.

Supplemental Figure 2. The impact of high-density lipoprotein on MACE risk in different subgroups of the population.

**Supplemental Figure legends**

**Supplemental Figure 1:**

**Title:** **HDL effect on mortality in different subgroups**

**Caption:** Supplemental Figure 1 illustrate examination of the relationship between HDL levels and the risks of mortality. In the variable column, "gender1" denotes female while "0" represents male. For other variables, "0" indicates absence of the medical history, and "1" indicates presence. In the table header, "0" corresponds to the L-HDL group and "1" to the H-HDL group. The HR (95% CI) column displays hazard ratios with 95% confidence intervals across patient subgroups. The dashed line signifies HR=1 (reference), while the solid line represents HR values with their 95% CIs. Lack of overlap between solid and dashed lines indicates statistically significant differences (P-values from regression analysis and p for interaction are reported).

**Supplemental Figure 2:**

**Title:** **HDL effect on MACE in different subgroups**

**Caption:** Supplemental Figure 2 illustrate examination of the relationship between HDL levels and the risks of MACE. In the variable column, "gender1" denotes female while "0" represents male. For other variables, "0" indicates absence of the medical history, and "1" indicates presence. In the table header, "0" corresponds to the L-HDL group and "1" to the H-HDL group. The HR (95% CI) column displays hazard ratios with 95% confidence intervals across patient subgroups. The dashed line signifies HR=1 (reference), while the solid line represents HR values with their 95% CIs. Lack of overlap between solid and dashed lines indicates statistically significant differences (P-values from regression analysis and p for interaction are reported).
